# Contrasting Hydraulic Architectures of Scots Pine and Sessile Oak at Their Southernmost Distribution Limits

**DOI:** 10.3389/fpls.2017.00598

**Published:** 2017-04-20

**Authors:** Elisabet Martínez-Sancho, Isabel Dorado-Liñán, Uwe G. Hacke, Hannes Seidel, Annette Menzel

**Affiliations:** ^1^Ecoclimatology, Department of Ecology and Ecosystem Management, Technische Universität MünchenFreising, Germany; ^2^Departamento de Silvicultura y Gestión de los Sistemas Forestales, Centro de Investigación Forestal–Instituto Nacional de Investigación y Tecnología Agraria y AlimentariaMadrid, Spain; ^3^Department of Renewable Resources, University of Alberta, EdmontonAB, Canada; ^4^Institute for Advanced Study, Technische Universität MünchenGarching, Germany

**Keywords:** anisohydric, drought, functional xylem anatomy, isohydric, hydraulic conductivity, Mediterranean Basin, *Pinus sylvestris*, *Quercus petraea*

## Abstract

Many temperate European tree species have their southernmost distribution limits in the Mediterranean Basin. The projected climatic conditions, particularly an increase in dryness, might induce an altitudinal and latitudinal retreat at their southernmost distribution limit. Therefore, characterizing the morphological and physiological variability of temperate tree species under dry conditions is essential to understand species’ responses to expected climate change. In this study, we compared branch-level hydraulic traits of four Scots pine and four sessile oak natural stands located at the western and central Mediterranean Basin to assess their adjustment to water limiting conditions. Hydraulic traits such as xylem- and leaf-specific maximum hydraulic conductivity (*K*_S-MAX_ and *K*_L-MAX_), leaf-to-xylem area ratio (*A*_L_:*A*_X_) and functional xylem fraction (FX) were measured in July 2015 during a long and exceptionally dry summer. Additionally, xylem-specific native hydraulic conductivity (*K*_S-N_) and native percentage of loss of hydraulic conductivity (PLC) were measured for Scots pine. Interspecific differences in these hydraulic traits as well as intraspecific variability between sites were assessed. The influence of annual, summer and growing season site climatic aridity (P/PET) on intraspecific variability was investigated. Sessile oak displayed higher values of *K*_S-MAX_, *K*_L-MAX_, *A*_L_:*A*_X_ but a smaller percentage of FX than Scots pines. Scots pine did not vary in any of the measured hydraulic traits across the sites, and PLC values were low for all sites, even during one of the warmest summers in the region. In contrast, sessile oak showed significant differences in *K*_S-MAX_, *K*_L-MAX_, and FX across sites, which were significantly related to site aridity. The striking similarity in the hydraulic traits across Scots pine sites suggests that no adjustment in hydraulic architecture was needed, likely as a consequence of a drought-avoidance strategy. In contrast, sessile oak displayed adjustments in the hydraulic architecture along an aridity gradient, pointing to a drought-tolerance strategy.

## Introduction

Drought is a key factor of tree species’ distribution in the Mediterranean Basin (Mitrakos, 1980; Cherubini et al., 2003), and climate projections for the next decades point to an increase in dryness in this region (Kirtman et al., 2013). For the most severe scenario of the last IPCC assessment report, an increase of ∼7°C in summer temperature and a decrease of ∼30% in April–September precipitation, as well as more intense summer droughts for the end of the 21st century are predicted (Collins et al., 2013). Climate change impacts may have global consequences in forest ecosystems, for instance, loss of ecosystem services (Anderegg et al., 2013); reduction of the terrestrial net productivity (Zhao and Running, 2010) and changes in sink–source carbon dynamics (Jones et al., 2009). In extreme cases, climate-change-induced negative effects can result in drought-induced tree mortality as reported worldwide by Allen et al. (2010).

Changes in climate may also extend the growing season due to higher spring temperatures (Menzel and Fabian, 1999; Menzel et al., 2006; Gordo and Sanz, 2010) and increase the water-use efficiency of the plants due to higher atmospheric CO_2_ concentrations (De Kauwe et al., 2013). However, in drought-prone areas such as the Mediterranean Basin where water availability is already limited (Giorgi and Lionello, 2008) and land-use changes are increasing the competition for this scarce resource (Ruiz-Benito et al., 2013), the above mentioned potential benefits have to be balanced against projected water limitations and their consequences on tree growth (Andreu-Hayles et al., 2011; Peñuelas et al., 2011).

Water loss through the stomata is an unavoidable consequence of carbon assimilation in plants. This loss must be compensated by water pulled upward from the roots to the leaves under negative hydrostatic pressure (Pockman et al., 1995). Under dry conditions, cavitation occurs inside the xylem conduits reducing the total hydraulic conductivity of the plant. Long and more severe water limitations lead to the collapse of the plant hydraulic system and ultimately, to tree death (McDowell, 2011). However, plants can partly cope with water limitations through physiological regulations in different parts of the soil–plant–atmosphere continuum (Sperry et al., 2002; Martínez-Vilalta et al., 2014), such as changes in the rooting system, in the hydraulic architecture, and/or in the regulation of transpirational water loss [e.g., by decreasing leaf-to-sapwood area ratio and/or increasing sensitivity of stomata to vapor pressure deficit (VPD)] (Tyree and Ewees, 1991; Oren et al., 1999; Martínez-Vilalta et al., 2004). Few studies have focused on the intraspecific variability of some of these hydraulic traits (Martínez-Vilalta et al., 2009; Benito-Garzón et al., 2011; Violle et al., 2014; Anderegg and Hillerislambers, 2016).

Many temperate European tree species reach their southernmost distribution limits in the Mediterranean region where they face suboptimal environmental conditions compared to the center of the distribution area (Hampe and Petit, 2005). However, the environmental conditions that allowed temperate species to establish in this region are changing (Giorgi and Lionello, 2008; Mariotti, 2010). There is now ample evidence that current climate change is promoting a rearrangement of the geographic distributions of plant and animal species world-wide (Parmesan and Yohe, 2003). Furthermore, Mediterranean plant species that have developed mechanisms in response to dry summers may be favored under future climate change scenarios and consequently, temperate species would either have to acclimate or to migrate to higher altitudes/latitudes (Peñuelas and Boada, 2003; Galiano et al., 2010). Understanding how hydraulic traits may respond to different climatic conditions will improve our knowledge on physiological limits of the temperate and boreal species and the heterogeneity in the response along their distribution range.

Scots pine (*Pinus sylvestris* L.) and sessile oak (*Quercus petraea* (Matt.) Liebl.) constitute two of the main tree species in Europe with contrasting hydraulic architecture as well as different ecophysiological strategies to deal with water limitations. Scots pine tends to avoid water stress by a strict stomata control, which is considered as an isohydric behavior (Irvine et al., 1998; Leo et al., 2014; Salmon et al., 2015). Under drought conditions, Scots pine adjusts its leaf-to-sapwood area ratio, leaf-specific hydraulic conductivity and total leaf area (Sterck et al., 2008; Martínez-Vilalta et al., 2009). In contrast, sessile oak usually maintains high transpiration and stomatal conductance (anisohydric behavior) under moderate drought conditions (Bréda et al., 1993b; Epron and Dreyer, 1993; Aranda et al., 2000; Klein, 2014). The projected changes in climate may shift the Mediterranean area beyond the ecological niche of these two species (Hampe and Petit, 2005; Lenoir et al., 2008). In this context, the differences in hydraulic architecture and strategy to cope with drought may be decisive for the capacity of resistance and resilience and hence, in future species persistence in the region.

In this study, we compared branch-level hydraulic traits of four natural pine and oak stands each, located at the southernmost limit of their distribution areas in the western and central Mediterranean Basin. Our objectives were (1) to characterize the interspecific differences due to the diverging physiological strategies (isohydric vs. anisohydric); (2) to assess intraspecific variability across the limit of the species’ distributions; and (3) to evaluate whether inter-site variability of hydraulic traits is linked to site aridity. Answering these questions would allow for a better understanding of the hydraulic strategies of the two species under natural conditions at the southernmost limits of the species distributions. The study was conducted during one of the warmest and driest summers in the region in the last six decades.

## Materials and Methods

### Study Species and Sites

Scots pine is an evergreen conifer with a wide distribution range across Eurasia. Its distribution is the largest of all species of the genus *Pinus*, and even of the whole *Pinaceae* family (Boratynski, 1991). This broad ecological range suggests a high degree of structural and/or functional plasticity. Pine wood is made by non-specialized tracheids that perform conductive and structural functions. In contrast, sessile oak is a deciduous temperate tree species predominantly distributed in central Europe. Oak wood is made up of vessels, tracheids, fibers, and parenchyma cells (Schweingruber, 1993). As a ring-porous species, the earlywood vessels support most of the water transport in the xylem, and in comparison to pine tracheids, they are by far more efficient in water transport (Tyree and Zimmermann, 2002).

**FIGURE 1:**
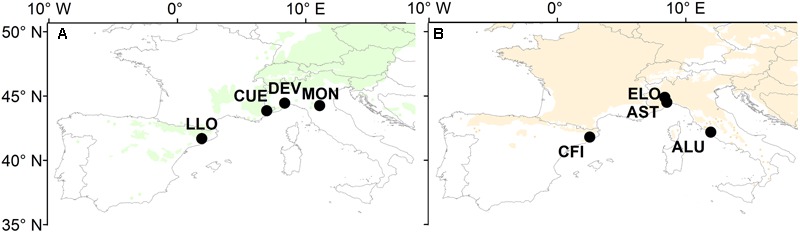
**Map of Europe showing the distribution of (A)** Scots pine and **(B)** sessile oak and the location of the eight study sites (for details of the study sites, see **Table 1**). Maps are a modified version from those available at www.euforgen.org.

Four natural stands of oak and four of pine were selected across the southernmost limits of both species distributions (**Figure 1**). The study sites cover a wide longitudinal area in the western and central part of the Mediterranean Basin (41.7° N – 44.9° N and 2.0° E – 11.9° E). They are located within the Mediterranean north and the Mediterranean mountains environmental zones (Metzger et al., 2005) characterized by a typical Mediterranean climate with a pronounced drop of precipitation during summer and one or two maxima of precipitation during the winter months.

For each site, annual sums of precipitation and mean annual temperature were extracted from the 0.25° gridded E-OBS dataset (Haylock et al., 2008) and are given as means in **Table 1** for the period 2011–2014, which also corresponds to the build-up time of the sampled branches. Mean annual temperatures ranged from 13.3°C to 15.5°C and 13.2°C to 16.1°C; and the annual sums of precipitation (P) ranged from 657.4 to 840.8 mm and 738.1 to 849.2 mm for the pine and oak sites, respectively. Potential evapotranspiration (PET) was calculated using the Hargreaves method (Hargreaves, 1994) included in the SPEI R package (Vicente-Serrano et al., 2010). Annual, summer and growing season P/PET and P-PET (climatic aridity indices) were calculated as proxies of mean potential drought stress for the studied period (Hogg, 1997; Knapp et al., 2008). However, results related to P-PET revealed identical patterns as P/PET and are not shown in this paper.

**Table 1:** Geographical characteristics of the study sites and mean climatic variables derived from E-OBS dataset for the studied period (2011–2014) corresponding to the build-up time of the sampled branches.

Site	Site code	Country	Species	LAT (°N)	LON (°E)	Elevation (m a.s.l.)	T (°C)	P (mm)	PET (mm)	P/PET	P/PET JJA	P/PET season
Sant Llorenç	LLO	Spain	*P. sylvestris*	41.722	1.997	500	15.5	599.6	970.4	0.62	0.22	0.49
Cuébris	CUE	France	*P. sylvestris*	43.894	7.064	720	13.4	840.8	820.3	1.02	0.26	0.61
Devia	DEV	Italy	*P. sylvestris*	44.489	8.460	364	14.0	781.5	728.1	1.07	0.27	0.64
Monte Sole	MON	Italy	*P. sylvestris*	44.293	11.181	560	13.3	657.4	933.5	0.70	0.22	0.48
Can Figueroles	CFI	Spain	*Q. petraea*	41.767	2.477	800	13.2	738.1	940.7	0.78	0.26	0.60
Sasselo	ELO	Italy	*Q. petraea*	44.478	8.479	445	14.0	781.5	728.2	1.07	0.27	0.64
Asti	AST	Italy	*Q. petraea*	44.855	8.323	330	14.1	849.2	873.8	0.97	0.40	0.66
Allumiere	ALU	Italy	*Q. petraea*	42.155	11.909	620	16.1	834.7	994	0.84	0.21	0.48

*LAT, latitude; LON, longitude; T, Mean annual temperature; P, sum annual precipitation; PET, potential evapotranspiration; P/PET, aridity index; P/PET JJA, summer aridity index, P/PET season, growing season aridity index.*

### Collection of Samples and Measurements

The field campaign took place during the last 2 weeks of July 2015, which was regionally (Supplementary Figure S1), and globally (NOAA National Centers for Environmental Information, 2016), an exceptionally hot period. At each site, one low branch per tree was collected from six trees with a throw saw. For technical reasons, distal segments from the selected branches (>50 cm in length) instead of the whole branches were collected. The samples were labeled and carefully wrapped in large plastic bags with wet towels to minimize dehydration. Additionally, diameter at breast height and total height of the sampled trees were measured (Supplementary Table S1). After collection, the samples were sent by courier to our laboratory at the TU Munich. All hydraulic measurements were conducted within 3 days after collection of the branches in the field. Once in the laboratory, the branches were successively cut under water by trimming the ends of each segment with a fresh razor blade. Five-year old internodes (4 years old plus the current growing season), located within the 50-cm distal segments, were selected for the measurements to obtain comparable results among trees and sites. Diameter and length of the resulting segments were recorded for each sample (see Supplementary Table S2 for mean dimensions).

In order to measure native hydraulic conductivity, the stem segments were fitted to a tubing apparatus (Sperry et al., 1988) filled with a filtered (0.2 μm) and degassed solution of 20 mM KCl and 1 mM CaCl_2_. The hydraulic conductivity (kg s^-1^m MPa^-1^) was calculated following the equation:

$$K_{\text{h}}=FL/\Delta P$$

where *F* is the flow rate (*F*, kg s^-1^), *L* is the length of the segment (m) and Δ*P* the driving force (MPa). The gravity-induced water flow rate through the segments was recorded every 10 s with an electronic balance (Mettler-Toledo XS204DR, Mettler-Toledo AG, Greifensee, Switzerland) interfaced with a computer. Stem hydraulic conductivity (*K*_h_/xylem area; kg m^-1^ s^-1^ MPa^-1^) was then calculated as the flow rate for a given pressure gradient and normalized by dividing the hydraulic conductivity by the total xylem area. Since we cannot exclude the possibility that oak internodes contained long vessels that embolised after collection, native conductivities (*K*_S-N_) are only reported for pine segments. In pine, the amount of native embolism (native percentage loss of hydraulic conductivity, PLC) was calculated as the percentage of *K*_S-N_ relative to *K*_S-MAX_. Native xylem embolism was reversed by applying two different methods for oak and pine, which were selected based in the results of previously performed tests. In the case of oak samples, branch segments were connected to a tubing system and flushed with the measuring solution for 1 h at 70 kPa. The pine samples were submerged in measuring solution and vacuum infiltrated for 1 h. Vacuum infiltration gave better results in pines, probably because it prevents the aspiration of the pit membranes (Schulte et al., 2015). Afterward, the maximum hydraulic conductivity was measured.

Leaf-specific maximum hydraulic conductivity (*K*_L-MAX_ = *K*_H-MAX_/leaf area; kg m^-1^ s^-1^ MPa^-1^) was calculated as the ratio of maximum hydraulic conductivity and the cumulative leaf area supplied by the segment. All the leaves distal to the 5-year-old segment later on used for the hydraulic measurements were collected and scanned (Epson Expression 10000 XL, Seiko Epson Corporation, Suwa, Japan). The total leaf area was measured using the image processing software Image J (Schneider et al., 2012). *K*_L-MAX_ is influenced by the leaf-to-xylem area ratio (*A*_L_:*A*_X_). Once the maximum hydraulic conductivity was determined, the stems were attached to a tubing system with a water reservoir and perfused with dye (0.1% crystal violet solution) to determine which parts of the xylem were functional after flushing or vacuum infiltration. Dye perfusions were conducted following the method described in Jacobsen et al. (2007). A pressure of ∼2 kPa was produced by lowering the water reservoir to 20 cm below the top of the dye solution, in order to stain the active xylem area. Cross sections were prepared from the center of each stem. The stained (functional) xylem area and the total xylem area (including non-stained xylem) were captured using a digital camera (Canon Rebel T2i, Canon, Krefeld, Germany) connected to a binocular microscope (Leica S6D, Leica camera AG, Wetzlar, Germany) and were measured with ImageJ (Schneider et al., 2012). Areas corresponding to the pith and the bark of the stems were excluded from the measurements when determining total xylem area and functional xylem area. The functional xylem fraction (FX) was expressed as the percentage of the ratio of the stained xylem area and the total xylem area. **Table 2** lists all branch-level traits with their respective units.

**Table 2:** Branch-level traits measured.

Variable	Acronym	Units
Xylem-specific native hydraulic conductivity	*K*_S-N_	kg m^-1^ s^-1^ MPa^-1^
Native percentage of loss hydraulic conductivity	PLC	%
Xylem-specific maximum hydraulic conductivity	*K*_S-MAX_	kg m^-1^ s^-1^ MPa^-1^
Leaf-specific maximum hydraulic conductivity	*K*_L-MAX_	10^-4^ kg m^-1^ s^-1^ MPa^-1^
Leaf-to-xylem area ratio	*A*_L_:*A*_X_	10^3^ m^2^ m^-2^
Functional xylem fraction	FX	%

### Data Analyses

Non-parametric approaches were chosen since the data were not normally distributed and could not be fit to a normal distribution by standard transformation techniques. Mann–Whitney (Wilcoxon) tests were performed to determine the significance of interspecific differences for all hydraulic traits. Pairwise Spearman’s rank correlations were performed to evaluate the relation among hydraulic traits within species. Kruskal–Wallis tests with a posterior Bonferroni corrected Mann–Whitney analyses were used to test differences in the hydraulic properties among sites for each tree species. In addition, linear regression analyses were used to relate site-specific means of hydraulic traits and annual, summer and growing season site aridity. Summer site aridity was considered as the ratio between precipitation and PET from June to August, whereas growing season aridity included the same parameters from March to October. Although the data were not normally distributed, parametric linear regressions were chosen because homoscedasticity and normality of errors of the models met the normality assumptions. The statistical software R (R Core Team, 2015) was used to perform the analyses.

## Results

### Interspecific Differences

**FIGURE 2:**
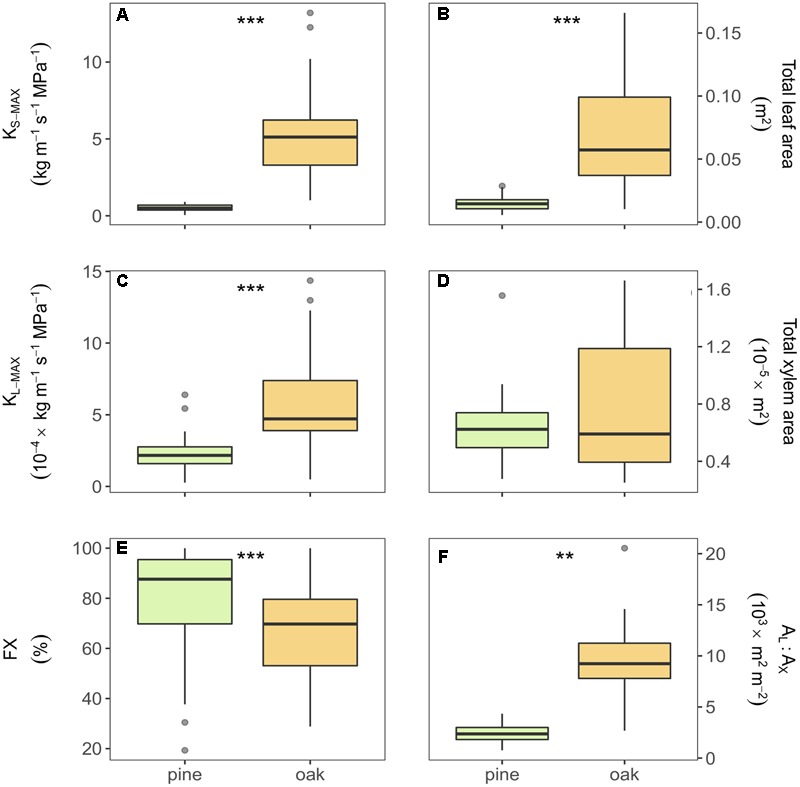
**Boxplots of the different hydraulic traits of Scots pine and sessile oak: (A)** xylem-specific maximum hydraulic conductivity, **(B)** total leaf area, **(C)** leaf-specific maximum hydraulic conductivity, **(D)** total xylem area, **(E)** functional xylem fraction, and **(F)** leaf-to-xylem area ratio. Boxes represent 50% of the data (between the first and third quartiles). Median is shown as horizontal line. Bars extend from the box to the highest/lowest value within 1.5 ^∗^ inter-quartile range (IQR). Points (outliers) are values > 1.5 ^∗^ IQR. *K*_S-MAX_, xylem-specific maximum hydraulic conductivity; *K*_L-MAX_, leaf-specific maximum hydraulic conductivity; *A*_L_:*A*_X_, leaf-to-xylem area ratio; FX, functional xylem fraction. ^∗∗^Indicate *p*-value < 0.01 and ^∗∗∗^indicate *p*-value < 0.001.

All measured hydraulic variables differed significantly between oak and pine samples (**Figure 2**). Compared to pine, oak displayed eight and three times higher xylem area- and leaf area-specific maximum hydraulic conductivity (K_S-MAX_ and K_L-MAX_), respectively. Oak branches showed six times higher values of total leaf area than pine branches, but similar values of total xylem area resulting in larger A_L_:A_X_ in oak branches than in pine (**Figures 2B,D,F**). Additionally, pine branches had a higher FX than oak branches (**Figure 2E**). Almost all xylem of the pine samples was stained (functional in water transport after vacuum infiltration) whereas in oak, the inner rings were not stained meaning that they were no longer functional in water transport.

### Inter-site Variability and Relation to Climate

**FIGURE 3:**
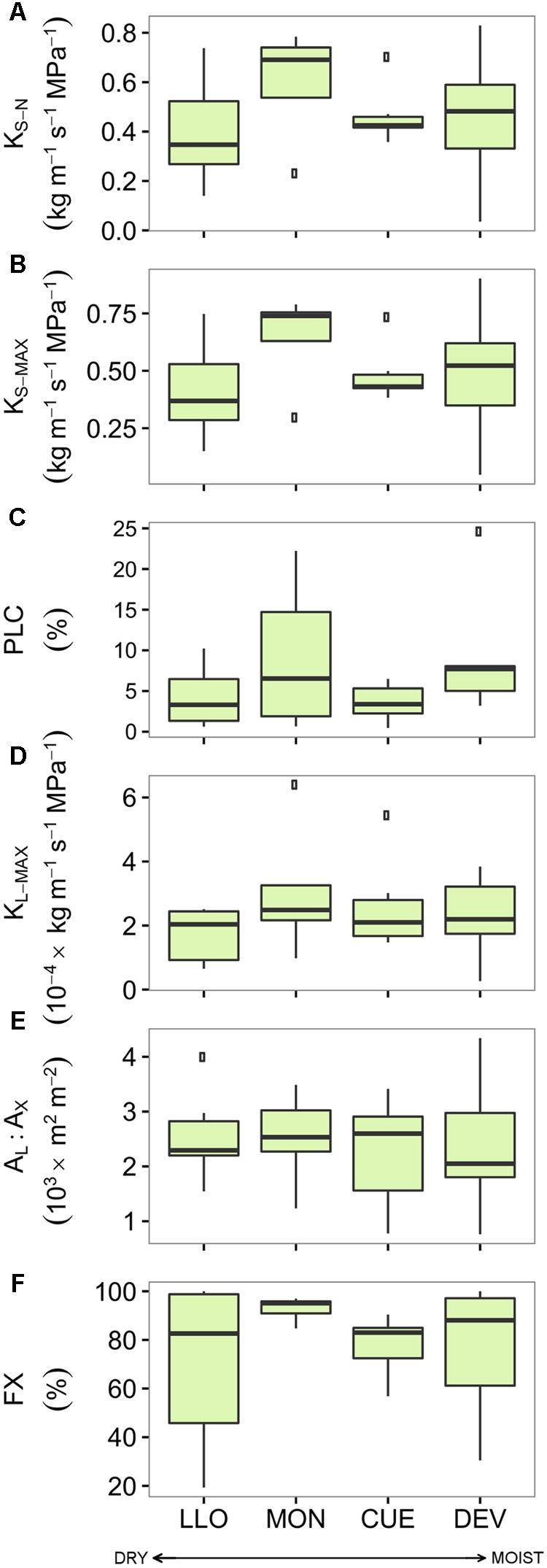
**Hydraulic traits of the four Scots pine populations sampled across an aridity gradient: (A)** xylem-specific native hydraulic conductivity, **(B)** xylem-specific maximum hydraulic conductivity, **(C)** native percentage of loss hydraulic conductivity, **(D)** leaf-specific maximum hydraulic conductivity, **(E)** leaf-to-xylem area ratio, and **(F)** functional xylem fraction. Sites are ordered respect to P/PET annual (see **Table 1**). Boxes represent 50% of the data (between the first and third quartiles). Median is shown as horizontal line. Bars extend from the box to the highest/lowest value within 1.5 ^∗^ inter-quartile range (IQR). Points (outliers) are values > 1.5 ^∗^ IQR. Note: None of the differences among sites was significant (see Supplementary Table S3). *K*_S-N_, xylem-specific native hydraulic conductivity; PLC, native percentage of loss hydraulic conductivity; *K*_S-MAX_, xylem-specific maximum hydraulic conductivity; *K*_L-MAX_, leaf-specific maximum hydraulic conductivity; *A*_L_:*A*_X_, leaf-to-xylem area ratio; FX, functional xylem fraction.

There were no significant differences in the studied hydraulic traits among pine sites (**Figure 3** and Supplementary Table S3). Branches from the Italian site MON displayed the highest values of *K*_S-N_, *K*_S-MAX_, *K*_L-MAX_, and FX while branches from LLO (the most arid site in Spain) showed the lowest values. All pine branches displayed low native PLC values. Branches from the French site CUE showed the lowest native mean PLC (3.59 %) while branches from MON showed the highest mean PLC (9.25 %). Consequently, no significant linear relation was found between the pine hydraulic traits and the annual, summer or growing season site aridity (**Table 3**).

**Table 3:** Statistics of the linear models to explain different hydraulic traits by annual, summer and growing season aridity index (P/PET) for the studied period (2011–2014) across the study sites.

		P/PET annual		P/PET summer		P/PET growing season	
Species		*R*^2^	*p*-value	*R*^2^	*p*-value	*R*^2^	*p*-value
Scots pine	*K*_S-N_	0.01	0.89	0.06	0.75	0.09	0.70
	PLC	0.01	0.89	0.01	0.92	<0.01	0.98
	*K*_S-MAX_	0.01	0.91	0.05	0.78	0.08	0.72
	*K*_L-MAX_	0.03	0.82	<0.01	0.97	<0.01	0.97
	*A*_L_:*A*_X_	0.84	0.08	0.77	0.12	0.10	0.69
	FX	0.01	0.88	0.06	0.74	0.77	0.12
Sessile oak	*K*_S-MAX_	0.99	<0.01	0.15	0.61	0.36	0.40
	*K*_L-MAX_	0.92	0.04	0.06	0.76	0.34	0.42
	*A*_L_:*A*_X_	0.71	0.15	0.02	0.85	0.05	0.78
	FX	0.8	0.11	0.56	0.25	0.69	0.17

*Summer aridity index considered values from June to August whereas the growing season aridity index considered values from March to October. *K*_*S-N*_, Xylem-specific native hydraulic conductivity; PLC, Native percentage of loss hydraulic conductivity; *K*_*S-MAX*_, Xylem-specific maximum hydraulic conductivity; *K*_*L-MAX*_, Leaf-specific maximum hydraulic conductivity; *A*_*L*_:*A*_*X*_, leaf-to-xylem area ratio; FX, functional xylem fraction.*

**FIGURE 4:**
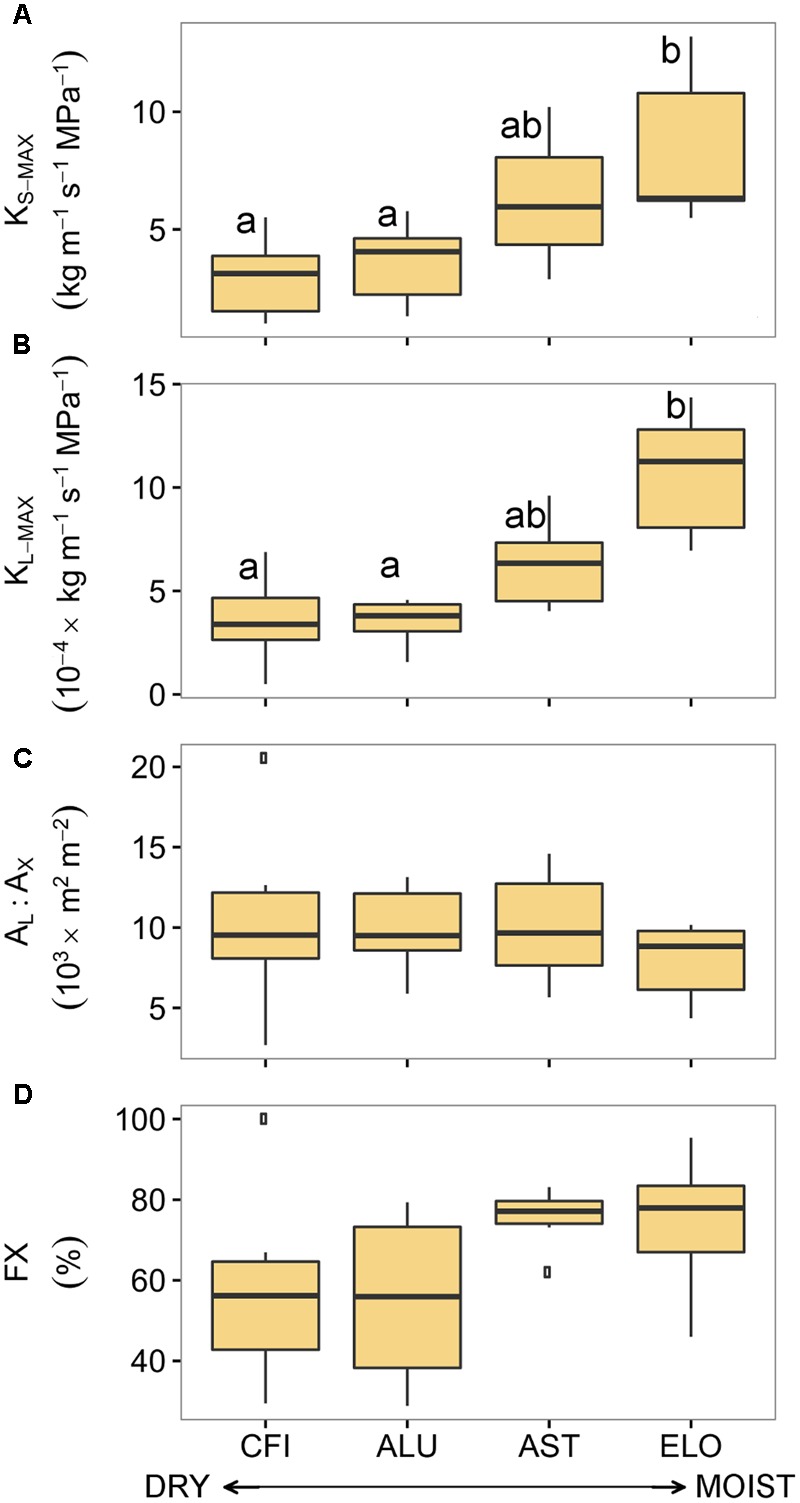
**Hydraulic traits of four sessile oak populations sampled across an aridity gradient: (A)** xylem-specific maximum hydraulic conductivity, **(B)** leaf-specific maximum hydraulic conductivity, **(C)** leaf-to-xylem area ratio, and **(D)** functional xylem fraction. Sites are ordered respect to P/PET annual (see **Table 1**). Boxes represent 50% of the data (between the first and third quartiles). Median is shown as horizontal line. Bars extend from the box to the highest/lowest value within 1.5 ^∗^ inter-quartile range (IQR). Points (outliers) are values > 1.5 ^∗^ IQR. Letters denote significant differences between sites (*p* < 0.05) (see Supplementary Table S4). *K*_S-MAX_, xylem-specific maximum hydraulic conductivity; *K*_L-MAX_, leaf-specific maximum hydraulic conductivity; *A*_L_:*A*_X_, leaf-to-xylem area ratio; FX, functional xylem fraction.

In contrast to the results obtained for pine, most of the oak hydraulic traits differed across an aridity gradient (**Figure 4**). In particular, traits such as *K*_S-MAX_ and *K*_L-MAX_ significantly differed (Supplementary Table S4). Branches from ELO (the least arid site in Italy) exhibited the highest values of *K*_S-MAX_ and *K*_L-MAX_ while the ones from CFI (the most arid site in Spain) showed the lowest conductivity. Although there were significant differences in total xylem area among oak stands (Supplementary Figure S2), no site-specific differences in A_L_:A_X_ were found. In addition, branches from the two less arid sites displayed higher fractions of functional xylem than branches from the two driest sites (**Figure 4D**).

These differences in hydraulic traits among oak sites were linked to the annual site aridity. Annual P/PET was positively related to *K*_S-MAX_ (*R*^2^= 0.99, *p* < 0.01), *K*_L-MAX_ (*R*^2^= 0.92, *p* < 0.04) and unrelated to *A*_L_:*A*_X_ and FX (*p* = 0.15 and *p* = 0.11, respectively; **Table 3**). However, none of the hydraulic traits were related to site-specific summer or growing season aridity.

### Relation among Hydraulic Traits

Branch hydraulic traits of pine samples were largely inter-correlated (Supplementary Figure S3). A highly significant positive correlation between *K*_S-N_ and *K*_S-MAX_ was found (*r* = 0.99, *p* < 0.001), as well as significant positive correlations of *K*_S-N_ and *K*_S-MAX_ with *K*_L-MAX_ and FX. Additionally, *K*_L-MAX_ showed a significant positive correlation with FX. However, *K*_L-MAX_ was the only trait showing a significant negative correlation with *A*_L_:*A*_X_ (*r* = -0.52, *p* < 0.05).

Four out of the six possible correlations of oak hydraulic traits were significant. A highly significant correlation was found between *K*_S-MAX_ and *K*_L-MAX_ (*r* = 0.84, *p* < 0.001). FX was positively correlated to *K*_S-MAX_ (*r* = 0.48, *p* < 0.05), *K*_L-MAX_ and *A*_L_:*A*_X_ (*r* = 0.38, *p* < 0.1 for both parameters).

## Discussion

### Ecophysiological Differences between Species

The differences between the branch-level hydraulic traits of oak and pine are likely the result of the contrasting ecophysiological and hydraulic strategies of both species. According to the results of our study sites located at the southernmost limits of the species’ distributions, oaks showed significantly higher hydraulic conductivities (*K*_S-MAX_ and *K*_L-MAX_) than pines (**Figure 2**). Generally, earlywood vessels of oak are more efficient conduits due to their lower hydraulic resistance than pine tracheids (Tyree and Zimmermann, 2002; Sperry, 2003; Sperry et al., 2006). Thus, even with a smaller share of FX, oak branches may transport more water per xylem unit under optimum conditions. Our results showed that oaks had a larger total leaf area (**Figure 2B**) and, consequently, oak leaves may have a better water supply in terms of *K*_L-MAX_ than pine needles (**Figure 2C**). In line with the more efficient xylem, higher *A*_L_:*A*_X_ ratios were found in oak (**Figure 2F**), suggesting that for the same xylem area, oak would be able to provide water to a larger leaf area than that of pines (e.g., Tyree and Zimmermann, 2002). However, these differences could be affected by the fact that the pine segments were longer than the maximum conduit length, and hence lumen and pit membrane hydraulic resistance was covered by our measurements; whereas some of the oak vessels are likely to be longer than the measured segments and thus basically only lumen resistance was measured. On the other hand and due to the longer mean internode’s length in oak branches (around 8 vs. 5, see Supplementary Table S2), vessels may have widened axially for a longer distance from the apex than pines (Anfodillo et al., 2013).

Differences in FX may be interpreted by the species-specific sensitivity to embolism (Hacke and Sperry, 2001). Large earlywood vessels of ring-porous species are more prone to embolisms caused by winter frosts or freeze-thaw cycles (Sperry et al., 1994; Hacke and Sauter, 1996; Tyree and Cochard, 1996; Davis et al., 1999). Consequently, oaks lose the main part of their hydraulic conductivity during winter time and recover it through newly produced earlywood vessels in the next spring (Cochard and Tyree, 1990; Cochard, 2006). Latewood vessels of oaks remain functional for some years providing a minimum flow even when the earlywood vessels might fail (Cochard and Tyree, 1990). In contrast, pine showed larger percentages of functional areas (FX). Due to the conservative strategy of Scots pine, several rings are functional and involved in water transport. For instance, a study reported that the annual build-up of new xylem tissue just accounts for 15–20% of the total hydraulic conductivity (Urli et al., 2013). The correlations among hydraulic traits were comparable for both species in terms of direction and significance, except for the relation between *A*_L_:*A*_X_ and *K*_L-MAX_. This relation was negative for both species, as expected (Mencuccini and Grace, 1995; Martínez-Vilalta et al., 2009). However, we did not find any significant relation between *A*_L_:*A*_X_ and *K*_S-MAX_ as reported in other studies for pine (Martínez-Vilalta et al., 2004, 2009).

### Trait Variation Related to Site Aridity

Pine did not show differences in any of the hydraulic traits across sites located at the southernmost edge of its distribution (**Figure 3**). Annual, summer and growing season site aridity were not linked to any hydraulic trait, although the range of mean P/PET values was wider for pine than for the oak sites. In contrast to our findings, Mencuccini and Grace (1995) suggested that *A*_L_:*A*_X_ in pine was influenced by differences in water VPD in the air, and pines growing at warmer sites produced less leaf area per unit of sapwood. This adjustment would maintain a constant water potential gradient within the stem at sites with different VPD and avoid exceedingly low water potentials. Similar results were found by Martínez-Vilalta et al. (2009) assessing the variability of branch-level hydraulic traits of pine across Europe. However, the authors showed a low variability in pine hydraulic traits since *A*_L_:*A*_X_, *K*_L-MAX_ and leaf carbon discrimination were the only traits out of eleven that were significantly associated with differences in moisture. Although our results did not reveal any significant differences in *A*_L_:*A*_X_ among sites, the range is in agreement with that reported by Martínez-Vilalta et al. (2009) for southern pine populations. Even though aridity varies across sites at the southernmost limits of the species’ distribution, the dominance of summer drought stress may force all trees to follow the same strategy. In fact, Stout and Sala (2003) found that intraspecific variability in *A*_L_:*A*_X_ is only detectable across larger geographic gradients.

Our results revealed low PLC values across all pine sites (**Figure 3C**). The small loss of conductivity, even in one of the warmest summers in the region in the last 65 years, might be a consequence of the conservative stomatal behavior common in pines (e.g., Poyatos et al., 2007) and other conifers (e.g., Anderegg and Hillerislambers, 2016). This early drought-response mechanism of isohydric species may reduce hydraulic loss but forces trees to rely on carbon reserves (McDowell, 2011; Salmon et al., 2015). Other studies carried out at the southernmost limits of the species distributions reported that prolonged drought periods could lead to a near-zero gas exchange compromising the carbon uptake during the main part of the growing season (Poyatos et al., 2013). Although the mechanisms behind the regulation of the non-structural carbohydrates are still not well understood (Sala et al., 2010), such a non-favorable situation would foster carbon starvation, or at least make the trees more vulnerable to biotic attacks (McDowell, 2011).

Our results clearly point to a covariation between oak hydraulic traits and site aridity (**Table 3**). Relatively anisohydric species, such as oak, are able to track environmental fluctuations in water potential (Martínez-Vilalta et al., 2014). The lower *K*_S-MAX_ and *K*_L-MAX_ values observed at the drier sites were not associated with changes in *A*_L_:*A*_X_ since the latter trait was similar across sites. Thus, part of the differences in conductivity among sites might be explained by changes in xylem traits such as smaller vessel diameters and/or more likely fewer functional vessels (**Figure 4D**). Earlywood vessel size is adjusted to drier conditions (Galle et al., 2010) and thus, might explain a larger portion of these differences in *K*_S-MAX_ and *K*_L-MAX_. Adjustments in xylem architecture but not in leaf traits were also found for *Quercus pubescens* growing under water limited conditions (Sterck et al., 2008). Additionally, higher values of *K*_L-MAX_ have been related to a greater photosynthetic capacity, which could be a potential benefit at wetter sites (Brodribb et al., 2002).

The drought tolerance of oak supports low water potentials under dry conditions (Bréda et al., 1993a; Gieger and Thomas, 2002; Urli et al., 2013). Moreover, many aspects of the water use strategy and whole-plant physiology are linked to the structure and function of the root system. In this case, oak has a deep rooting system (Bréda et al., 1993b) and under dry conditions increases the proportion of fine-root biomass relative to leaf biomass (Bréda et al., 1995; Gieger and Thomas, 2002). This reinforces the relatively anisohydric strategy of oak during dry periods. Thus, carbon uptake may not be compromised under moderate drought conditions (Li et al., 2013). The accumulation of reserves during the growing season is essential for oak since the growth of new earlywood vessels will rely on the stored carbohydrates to restore the water pathway (Barbaroux and Bréda, 2002; Michelot et al., 2012). Moreover, and despite our limited understanding of xylem refilling (Sperry, 2013), part of the stored carbohydrates could be used to refill embolized xylem (Salleo et al., 1996), as found in a congeneric *Quercus* species (Taneda and Sperry, 2008). Our results suggests that oak is able to adjust the xylem architecture to site aridity.

However, recent studies highlighted the importance of the conduits axially increasing trend on hydraulic conductivity measurements (Petit et al., 2016). This axial conduit widening is a universal configuration of the xylem architecture (Anfodillo et al., 2013; Olson et al., 2014), and predicts that conduits should widen from stem/branch tip to the base, and, consequently also increases the theoretical hydraulic conductivity. In our case, the mean difference in the 5-year-old internode length among sites was 1 cm for pines and 2.7 cm for oaks (Supplementary Table S2), resulting in none significant differences in segment length among sites for both studied species (*p* > 0.05 for Krukal–Wallis test) (Supplementary Figure S4). Consequently, the effect of the path length on vessel size and therefore on the hydraulic conductivity, could be considered negligible in our specific case.

It should also be remarked that the characterization of site aridity in our study was just based on climatic variables. Although differences among sites are largely triggered by climatic conditions, other factors such as soil characteristics may also matter. On the other hand, water potentials could not be measured in the field during the sampling campaign and, consequently, we could not quantify the actual tension within the xylem during the extraordinary dry and hot spell of July 2015. The results shown here could be complemented with experiments under controlled conditions (i.e., dry-down experiments) to make more robust statements.

### Future Perspectives

Most tree species operate with narrow hydraulic safety margins, which render them vulnerable to hydraulic failure (Choat et al., 2012). However, there are clear differences between angiosperms and gymnosperms (Johnson et al., 2012; Carnicer et al., 2013). Even taking into consideration the water potential inducing a 88% loss of stem conductivity (P88) to calculate these margins as recently proposed by Urli et al. (2013), conifers follow a safer strategy and show wider hydraulic safety margins than angiosperms.

The lack of variability in hydraulic traits across Scots pine populations found in this study suggests that this species performed a drought-avoidance survival strategy by closing stomata rather than investing in optimizing the hydraulic architecture to the harsh environmental conditions. This conservative strategy might compromise carbon gain during prolonged drought periods but, at the same time, preserves the integrity of the hydraulic system by avoiding possible damages during extreme events. On the other hand, the observed variability in the sessile oak hydraulic traits suggests that they are able to adjust to different levels of aridity. However, and despite the difficulty of measuring the loss of conductivity in oaks under natural conditions, such a drought-tolerance strategy might imply possible hydraulic failures under extreme climatic events.

A recent study on hydraulic traits at the dry-range limit in two of the most widely distributed species in North America, ponderosa pine (*Pinus ponderosa*) and trembling aspen (*Populus tremuloides*), revealed similar patterns (Anderegg and Hillerislambers, 2016). The authors suggested that the drought-avoidance strategy performed by ponderosa pine may lead to a range retreat as a response to a long-term drying trend. In contrast, trembling aspen performed a more drought-tolerant strategy by constructing a safer xylem, although such a hydraulic strategy may turn individuals more vulnerable to extreme events. Given the projected future drying of the Mediterranean Basin, impacts on pine and oak might be related to the intensity and the duration of drought events. Our results suggest that sessile oak holds the capacity to plastically adjust its hydraulic architectures to dryness, whereas Scots pine does not have such adaptable hydraulic structure, having both strategies different advantages but also disadvantages. Although recent publications have highlighted the ongoing replacement of pine by oak at the lower edge of the elevation range in some regions of the Mediterranean basin and inner alpine dry valleys (Galiano et al., 2010; Rigling et al., 2013; Vayreda et al., 2016), the intensity, duration and recurrence of forthcoming dry spells might be a crucial factor shaping the southernmost distribution limits of both tree species.

## Author Contributions

AM, ID-L, UH, HS, and EM-S conceived the ideas. ID-L and EM-S collected the samples. EM-S and HS carried out the analyses with help from UH. The writing of the article was led by EM-S and many contributions and comments were made by ID-L, UH, HS and AM.

## Conflict of Interest Statement

The authors declare that the research was conducted in the absence of any commercial or financial relationships that could be construed as a potential conflict of interest.
