# Supplementary material for: Contrasting Hydraulic Architectures of Scots Pine and Sessile Oak at Their Southernmost Distribution Limits
Source: Front Plant Sci. 2017 Apr 20;8:598. doi: 10.3389/fpls.2017.00598 (PMC5397420; doi:10.3389/fpls.2017.00598)
Supplement: Supplementary file 1 [file Data_Sheet_1.docx]

Supplementary Material

Contrasting hydraulic architectures of Scots pine and sessile oak at their southernmost distribution limits

Elisabet Martínez-Sancho^*^, Isabel Dorado-Liñán, Uwe G. Hacke, Hannes Seidel, Annette Menzel

*** Correspondence:** Elisabet Martínez-Sancho [martinez@wzw.tum.de](mailto:martinez@wzw.tum.de)
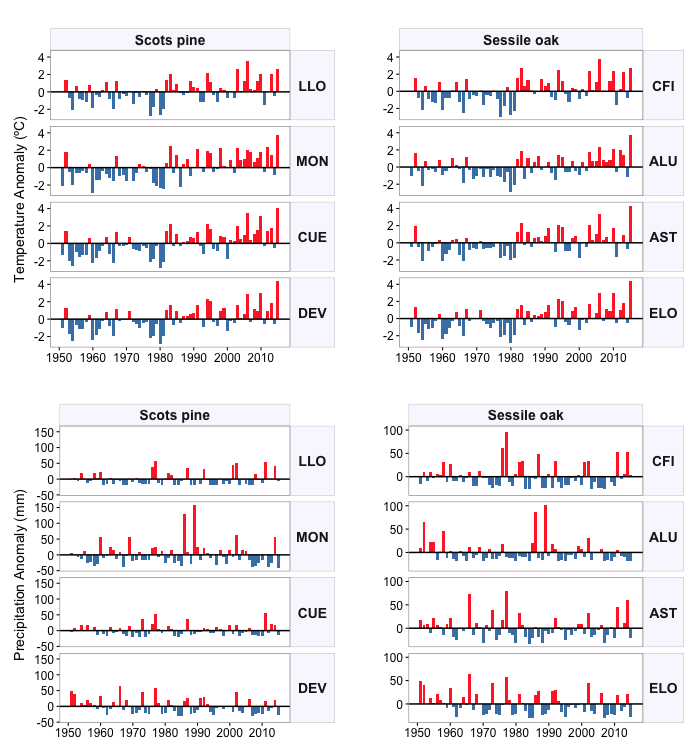


**Supplementary Figure S1.** July temperature and precipitation anomalies for the period 1951-2015 derived from gridded E-OBS database.

**
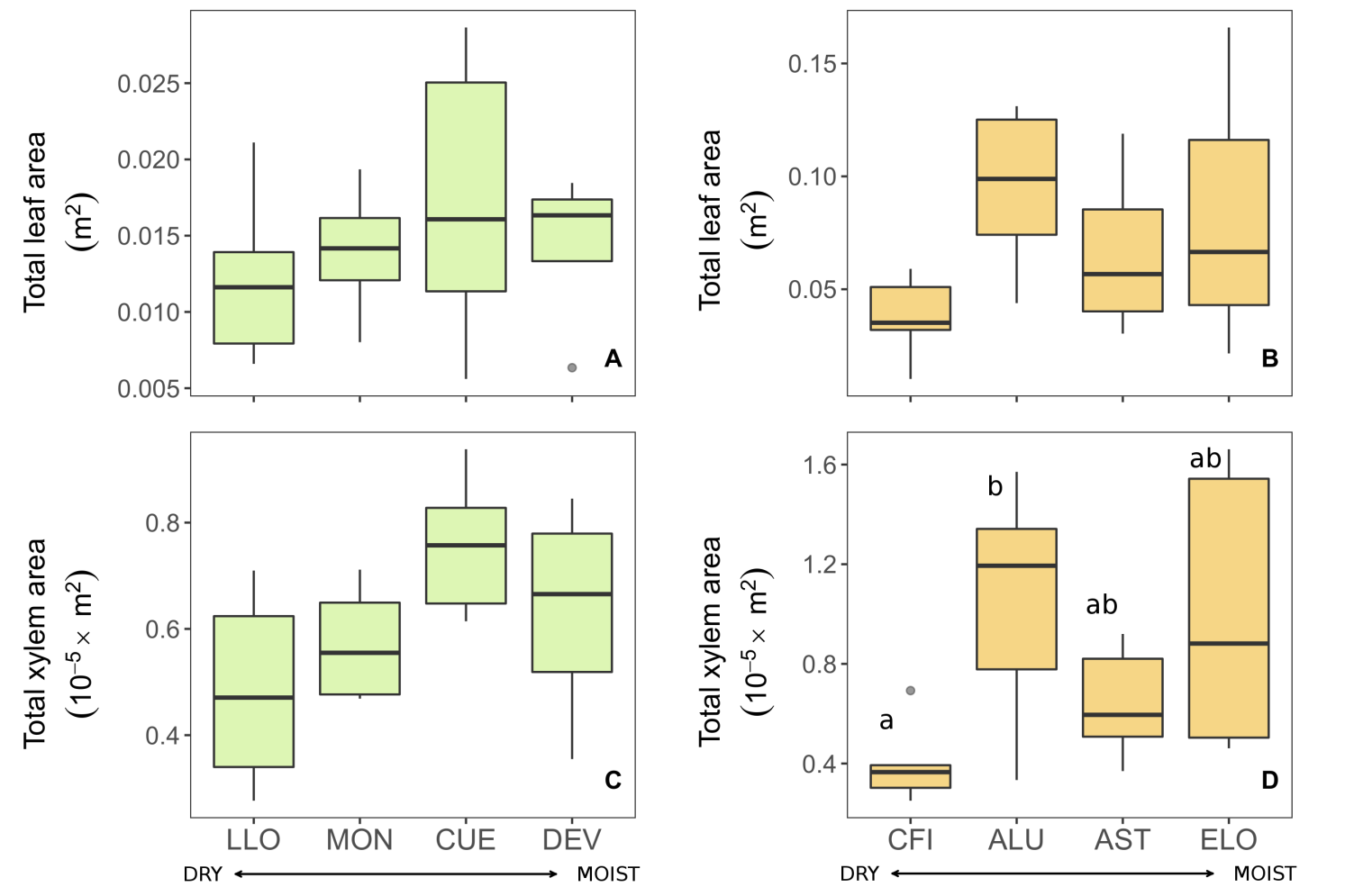
**

**Supplementary Figure S2.** Total leaf area and total xylem area of the four Scots pine (A, C) and the four sessile oak (B, D) populations sampled across an aridity gradient. Sites are ordered according to the annual P/PET (see Table 1). Boxes represent 50% of the data (between the first and third quartiles). Median is shown as horizontal line. Bars extend from the box to the highest/ lowest value within 1.5 * inter-quartile range (IQR). Points (outliers) are values > 1.5 * IQR. Note: Letters denote significant differences between sites (*p*< 0.05) (see Supplementary Table S3 and S4).


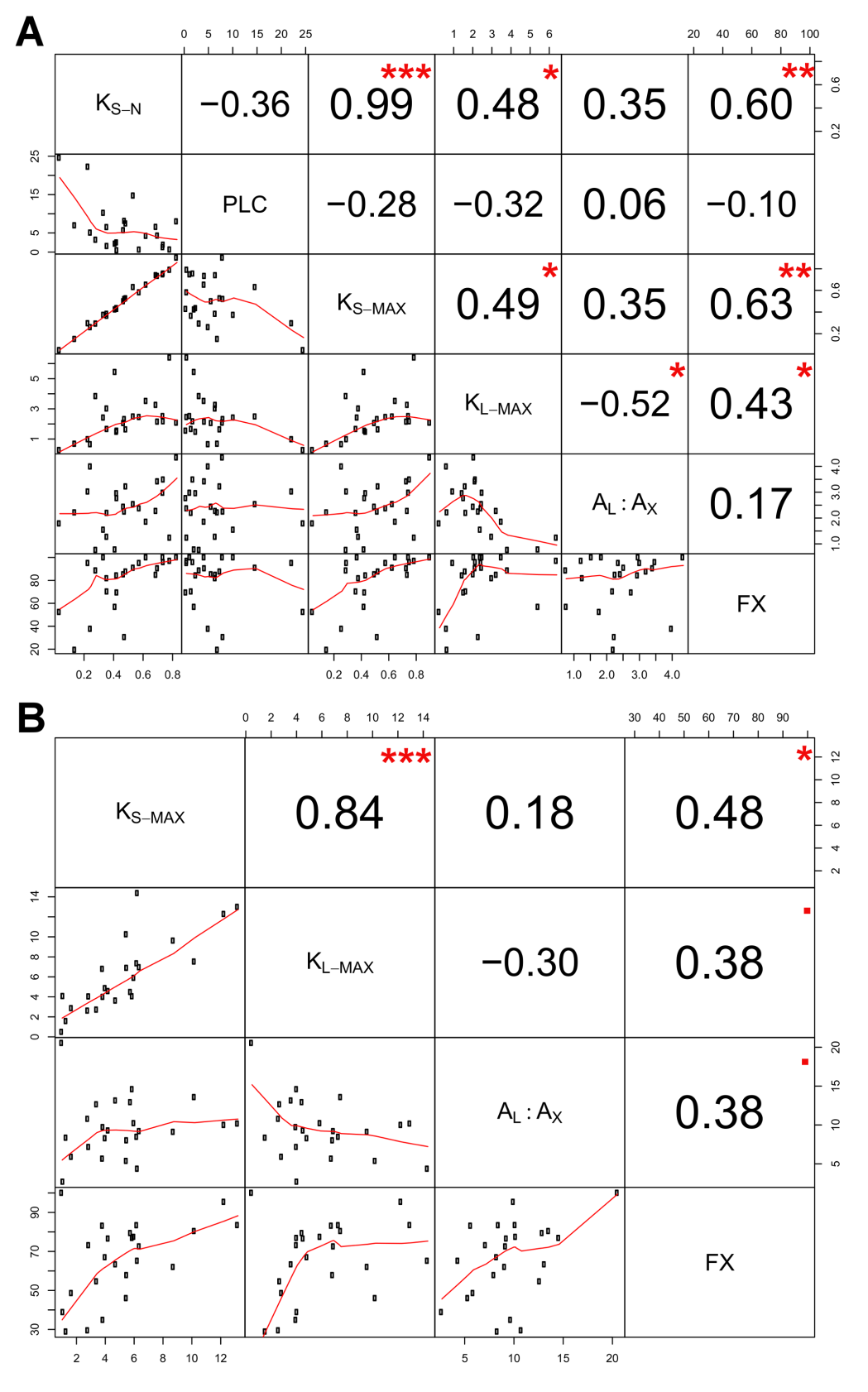


**Supplementary Figure S3.** Matrix of pairwise Spearman’s rank correlation among hydraulic traits of (A) Scots pine and (B) sessile oak. The names of the variables on the diagonal refer to both rows and columns (for abbreviations see Table 2). Lower triangle boxes contain miniature scatterplots of the two variables. Upper triangle boxes display the corresponding correlation coefficients with the significance levels. ·, *, **, *** indicate *p*-value < 0.1, 0.05, 0.01 and 0.001, respectively.

**
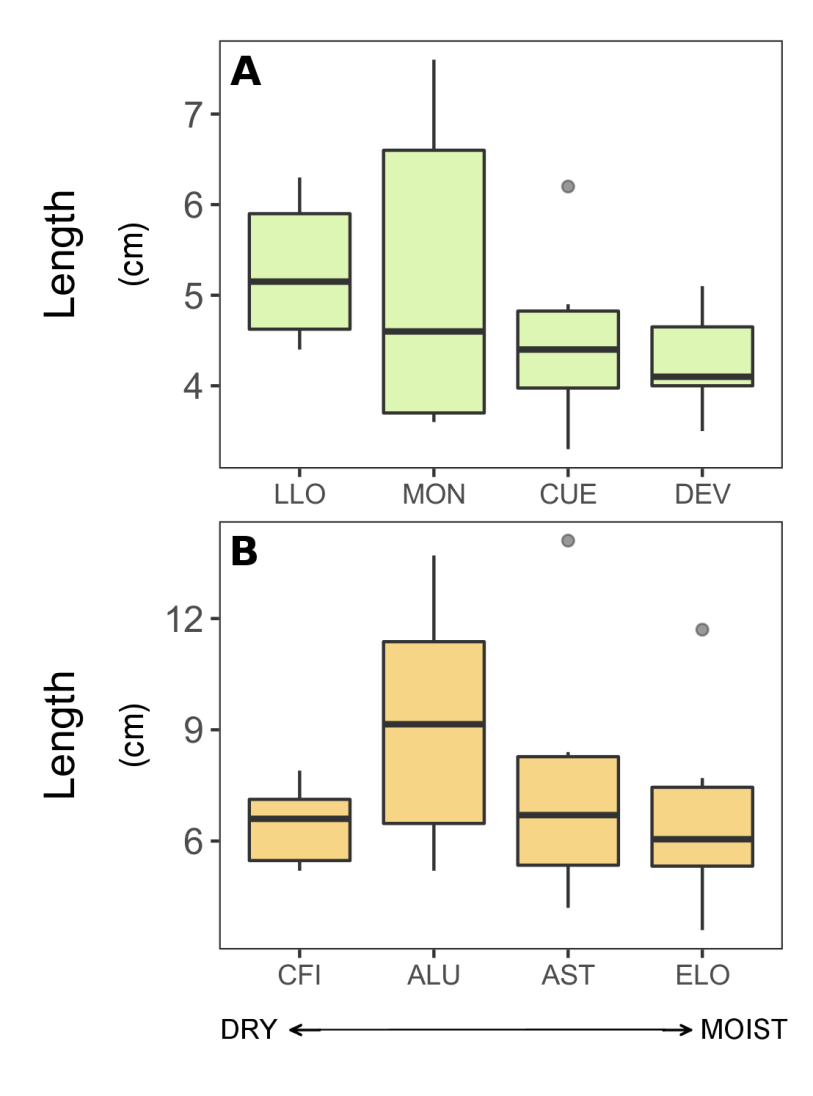
**

**Supplementary Figure S4.** Length of the 5-year old internode segments of the four Scots pine (A) and the four sessile oak (B) populations sampled across an aridity gradient. Sites are ordered according to annual P/PET (see Table 1). Boxes represent 50% of the data (between the first and third quartiles). Median is shown as horizontal line. Bars extend from the box to the highest/ lowest value within 1.5 * inter-quartile range (IQR). Points (outliers) are values > 1.5 * IQR. Note: No differences were fond across sites for the two studied species (Kruskal-Wallis test *p*> 0.05).

**Supplementary Table S1.** Characteristics of the sampled trees (n=6) as mean value ± standard deviation per site of diameter at breast height (DBH) and height.

| SPECIES | SITE | DBH (cm) | HEIGHT (m) |
| --- | --- | --- | --- |
| Scots pine | LLO | 8.27 ± 4.60 | 5.87 ± 2.11 |
|  | MON | 24.92 ± 4.16 | 8.99 ± 1.67 |
|  | CUE | 24.43 ± 6.00 | 5.49 ± 1.89 |
|  | DEV | 15.08 ± 8.87 | 4.55 ± 1.85 |
| Sessile oak | CFI | 27.38 ± 7.51 | 13.25 ± 1.21 |
|  | ALU | 29.02 ± 18.04 | 12.04 ± 3.30 |
|  | AST | 14.60 ± 10.02 | 9.03 ± 4.23 |
|  | ELO | 14.83 ± 6.36 | 9.86 ± 2.39 |

**Supplementary Table S2.** Dimensions of the 5-year old internode segments as mean value ± standard deviation (n=6) per site.

| SPECIES | SITE | DIAMETER (mm) | LENGTH (cm) |
| --- | --- | --- | --- |
| Scots pine | LLO | 3.75 ± 0.49 | 5.27 ± 0.80 |
|  | MON | 4.17 ± 0.52 | 5.22 ± 1.79 |
|  | CUE | 4.91 ± 0.40 | 4.52 ± 0.99 |
|  | DEV | 4.42 ± 0.44 | 4.27 ± 0.58 |
| Sessile oak | CFI | 3.57 ± 0.55 | 6.45 ± 1.09 |
|  | ALU | 4.92 ± 1.02 | 9.15 ± 3.36 |
|  | AST | 4.12 ± 0.63 | 7.57 ± 3.58 |
|  | ELO | 4.91 ± 1.55 | 6.73 ± 2.80 |

**Supplementary Table S3.** Hydraulic traits of the four Scots pine sites as mean value ± standard deviation (n = 6) per site and corresponding *p*-value of Kruskal-Wallis tests. K_S-N_, xylem-specific native hydraulic conductivity; PLC, native percentage of loss hydraulic conductivity; K_S-MAX_, xylem-specific maximum hydraulic conductivity; K_L-MAX_, leaf-specific maximum hydraulic conductivity; A_L_:A_X_, leaf-to-xylem area ratio; FX, functional xylem fraction; A_L_, total leaf area; A_X_, total xylem area.

|  |  | LLO | MON | CUE | DEV | *p*-value |
| --- | --- | --- | --- | --- | --- | --- |
| K_S-N_ | kg m^–1^ s^–1^ MPa^–1^ | 0.40 ± 0.22 | 0.60 ± 0.23 | 0.47 ± 0.12 | 0.46 ± 0.27 | 0.137 |
| K_S-MAX_ | kg m^–1^ s^–1^ MPa^–1^ | 0.41 ± 0.22 | 0.64 ± 0.20 | 0.48 ± 0.13 | 0.49 ± 0.29 | 0.299 |
| PLC | % | 4.28 ± 3.82 | 9.20 ± 9.13 | 3.59 ± 2.29 | 9.25 ± 7.79 | 0.252 |
| K_L-MAX_ | 10^-4^ x kg m^–1^ s^–1^ MPa^–1^ | 1.73 ± 0.88 | 3.05 ± 2.04 | 2.61 ± 1.49 | 2.27 ± 1.30 | 0.689 |
| A_L_:A_X_ | 10^3^ x m^2^ m^-2^ | 2.55 ± 0.84 | 2.51 ± 0.85 | 2.27 ± 1.03 | 2.37± 1.25 | 0.948 |
| FX | % | 70.37 ± 34.76 | 92.70 ± 5.00 | 77.96 ± 12.51 | 76.50 ± 20.54 | 0.404 |
| A_L_ | m^2^ | 0.012 ± 0.005 | 0.014 ± 0.004 | 0.017 ± 0.009 | 0.015 ± 0.006 | 0.692 |
| A_X_ | 10^-5^ x m^2^ | 0.48 ± 0.18 | 0.57 ± 0.11 | 0.75 ± 0.13 | 0.77 ± 0.42 | 0.070 |
|  |  |  |  |  |  |  |

**Supplementary Table S4.** Hydraulic traits of the four sessile oak sites as mean value ± standard deviation (n = 6) per site and corresponding *p*-value of Kruskal-Wallis tests. Letters label homogeneous groups within a variable based on a Mann-Whitney test. K_S-MAX_, xylem-specific maximum hydraulic conductivity; K_L-MAX_, leaf-specific maximum hydraulic conductivity; A_L_:A_X_, leaf-to-xylem area ratio; FX, functional xylem fraction; A_L_, total leaf area; A_X_, total xylem area.

|  |  | CFI | ALU | AST | ELO | *p*-value |
| --- | --- | --- | --- | --- | --- | --- |
| K_S-MAX_ | kg m^–1^ s^–1^ MPa^–1^ | 2.98 ± 1.74 a | 3.60 ± 1.76 a | 6.26 ± 2.86 ab | 8.30 ± 3.46 b | 0.010 |
| K_L-MAX_ | 10^-4^ x kg m^–1^ s^–1^ MPa^–1^ | 3.60 ± 2.19 a | 3.51 ± 1,14 a | 6.31 ± 2.15 ab | 10.69 ± 3.05 b | 0.002 |
| A_L_:A_X_ | 10^3^ x m^2^ m^-2^ | 10.49 ± 5.96 | 9.88 ± 2.77 | 10.05 ± 3.51 | 7.92 ± 2.47 | 0.716 |
| FX | % | 57.93 ± 24.65 | 55.25 ± 21.25 | 75.48 ± 7.43 | 74.31 ± 17.31 | 0.150 |
| A_L_ | m^2^ | 0.038 ± 0.018 | 0.095 ± 0.035 | 0.065 ± 0.034 | 0.081 ± 0.056 | 0.08 |
| A_X_ | 10^-5^ x m^2^ | 0.39 ± 0.16 | 1.05 ± 0.47 | 0.64 ± 0.22 | 1.00 ± 0.58 | 0.04 |
